# The Effects of the COVID-19 Pandemic on Age-Based Disparities in Digital Health Technology Use: Secondary Analysis of the 2017-2022 Health Information National Trends Survey

**DOI:** 10.2196/65541

**Published:** 2024-12-04

**Authors:** Yuanbo Qiu, Huang Huang, Junjie Gai, Gianluca De Leo

**Affiliations:** 1 School of Journalism and Communication South China University of Technology Guanzhou China; 2 Department of Health Management, Economics, and Policy School of Public Health Augusta University Augusta, GA United States; 3 Department of Health Management and Policy College of Public Health University of Iowa Iowa city, IA United States

**Keywords:** age-based disparities, health equity, digital health technology use, digital divide, health policy, COVID-19, mobile phone

## Abstract

**Background:**

The COVID-19 pandemic accelerated the adoption of digital health technology, but it could also impact age-based disparities as existing studies have pointed out. Compared with the pre-pandemic period, whether the rapid digitalization of the health care system during the pandemic widened the age-based disparities over a long period remains unclear.

**Objective:**

This study aimed to analyze the long-term effects of the COVID-19 pandemic on the multifaceted landscape of digital health technology used across diverse age groups among US citizens.

**Methods:**

We conducted the retrospective observational study using the 2017-2022 Health Information National Trends Survey to identify the influence of the COVID-19 pandemic on a wide range of digital health technology use outcomes across various age groups. The sample included 15,505 respondents, which were categorized into 3 age groups: adults (18-44 years), middle-aged adults (45-64 years), and older adults (more than 65 years). We also designated the time point of March 11, 2020, to divide the pre- and post-pandemic periods. Based on these categorizations, multivariate linear probability models were used to assess pre-post changes in digital health technology use, controlling for demographic, socioeconomic, and health-related variables among different age groups.

**Results:**

Essentially, older adults were found to be significantly less likely to use digital health technology compared with adults, with a 26.28% lower likelihood of using the internet for health information (*P*<.001) and a 32.63% lower likelihood of using health apps (*P*<.001). The usage of digital health technology for all age groups had significantly increased after the onset of the pandemic, and the age-based disparities became smaller in terms of using the internet to look for health information. However, the disparities have widened for older adults in using the internet to look up test results (11.21%, *P*<.001) and make appointments (10.03%, *P*=.006) and using wearable devices to track health (8.31%, *P*=.01).

**Conclusions:**

Our study reveals a significant increase in the use of digital health technology among all age groups during the pandemic. However, while the disparities in accessing online information have narrowed, age-based disparities, particularly for older adults, have widened in most areas such as looking up test results and making appointments with doctors. Therefore, older adults are more likely left behind by the rapidly digitalized US health care system during the pandemic. Policy makers and health care providers should focus on addressing these disparities to ensure equitable access to digital health resources for US baby boomers.

## Introduction

Digital health technology use is the usage of information and communication technologies such as mobile phones, computers, and wearable devices to engage individuals in health-related activities, including seeking health information, reviewing test results, scheduling appointments, and monitoring health conditions [1]. Digital health technology facilitates health information seeking and health management, and has become increasingly vital for individuals to access health information and remote care in recent decades [2,3]. The adoption and implementation of digital health technology in the US health care system have been accelerated dramatically during the COVID-19 pandemic when individual mobility and in-person contacts were greatly restricted by social distancing and quarantine orders [4,5]. Researchers record approximately 50% of the US citizens have embraced smartphones, computers, or other electronic devices to engage with health apps, track personal health metrics, and consult with health care professionals to various degrees during the pandemic [6,7].

Despite the potential benefits, heavy reliance on digital health technology may inadvertently intensify disparities in access to health information and resources across different demographic groups [8-10]. The digital divide, characterized by unequal access to digital technology in health care, was found to exist for decades and has been intensified during the pandemic due to various barriers, including disparities in internet access [11,12], broadband infrastructure [13], or digital literacy skills [14]. Consequently, the widening digital divide may render vulnerable populations less likely to access health care services and more susceptible to diseases, therefore amplifying health inequalities [15,16].

The age-based digital divide, or the gray digital divide, constitutes a significant challenge for older adults in receiving health information, accessing health services, and managing health conditions in the information era. The low adoption and limited use of digital health technology by older adults, in contrast to younger generations, have been well documented over a long period [17-19]. Entering the pandemic, older adults’ greater needs for regular health consultations were constrained because of the restrictions on physical interaction, and therefore, accessing health care services remotely through digital health technology became the primary or even the only option [20]. The rapid advancement of digital health technology and infrastructure during the pandemic significantly increased telehealth use among the older adult population [21]. This trend seems to echo the observations regarding the narrowing race and ethnicity disparity in the use of digital health technology: the threat of a global pandemic and the fear of disease and death, or in other words, the coronavirus nosophobia, overpowered objective or subjective barriers of using updated digital health technology among the vulnerable populations [22].

Nevertheless, researchers also warned that the surging demands of digital health technology generated by the pandemic among older adults have not been adequately catered [23]. As several preexisting disadvantages such as the lack of digital skills, economic disparities, technological anxiety, and distrust of digital health care remained the key factors that contributed to age-based disparities [20,24,25], the older groups encountered more digital obstacles and experienced more digital isolation compared with their younger counterparts during the pandemic [26,27]. In such circumstances, failing to effectively use digital health technology may result in inadequate chronic disease management, delayed health care access, difficulty in seeking health information, disengagement in health behaviors or promotion efforts, and ultimately worse health outcomes for older generations [28-30]. If the age-based disparity in the use of digital health technology continues to widen during the COVID-19 period, the older generations left behind will inevitably become marginalized by the accelerated digitalization of the US health care system. Therefore, as Healthy People 2030 highlights the critical roles of health information techniques in health communication and promotion [31], whether the COVID-19 pandemic has narrowed or widened the age-based disparities in the long term becomes a pressing concern of policy makers and scholars.

Despite the increased significance and ample evidence of age-based disparities in the use of digital health technology before or at the onset of the pandemic, a question remains unanswered: to what extent has the age-based digital divide been reshaped by the pandemic over a long term? Researchers tend to theoretically discuss the reasons, potential harms, or feasible strategies of general disparity in digital health technology usage [12,32,33]. However, research gaps persist, including restricted study periods, lack of prepandemic baseline comparison, and small sample size. Thus, there is a pressing need for empirical evidence on the changes in digital health technology use across different generations throughout the COVID-19 pandemic. Answering this question not only helps us identify specific underused digital health technology and often-neglected vulnerable age groups at the present but also informs effective public health communication and intervention strategies for the underserved and vulnerable groups in the future.

To address this gap, we leverage the multivariate linear probability model (LPM) to analyze the large, nationwide representative sample in the Health Information National Trends Survey (HINTS) from 2017 to 2022. Our investigation delves into the multifaceted landscape of digital health technology use across diverse age groups and covers a long period from the prepandemic period, the pandemic’s onset, to the late pandemic period. The findings provide empirical evidence of age-based disparities changed by the pandemic and justify further solutions and interventions to mitigate age-based disparities in digital health technology usage among the baby boomer generation.

## Methods

### Data

We conducted the retrospective observational study using the 2017-2022 HINTS to identify the influence of the COVID-19 pandemic on a wide range of digital health technology uses across various age groups. HINTS is a nationally representative survey administered by the National Cancer Institute (NCI) every 1 or 2 years since 2003. It targets the US civilian noninstitutionalized adults aged 18 years and older [34]. In order to guarantee a nationally representative sample, HINTS adopts a 2-stage sampling design that first generates a random stratified household address sample from the sampling frame and randomly selects one adult in the household to complete the survey. It provides detailed information including but not limited to health information searching, medical health records, health behaviors, health care access, health beliefs, and demographics, which enables health scholars to better monitor the changes in health-related knowledge and health information technology usage among the US public.

We constructed the pooled cross-sectional dataset (N=22,344) from the HINTS 5 cycles 1 to 4 (2017-2020) and HINTS 6 (2022) original datasets. A special case is that HINTS divides the 2020 sample into the pre- and post-pandemic–onset periods based on the indicator of whether the respondents completed the survey after March 11, 2020 [12]. However, because HINTS asked about the respondents’ digital health technology use in the past 12 months, the records of the 2020 post-pandemic–onset sample may potentially reflect their use before the pandemic, so we decided to exclude the 2020 post-pandemic–onset sample from our analytic sample (n=2428). After listwise deletion of all observations with any missing values in the control covariates (n=4411), the final sample size was 15,505; however, due to the change of survey design and missing values in outcome variables, readers should notice the sample sizes in regression results vary by outcomes.

### Measurement

#### Outcomes

Our outcomes of digital health technology use involved using the internet, health apps in mobile devices, and electronic wearable devices to conduct health-related activities, consistent with previous studies [18,35,36]. First, the outcome of internet use asked whether the respondents have ever used the internet to (1) look for health or medical information, (2) send a message to a health care provider or a health care provider’s office, (3) view medical test results, or (4) make an appointment with a health care provider. Second, health app usage focused on whether respondents ever used the health or wellness app on a tablet or smartphone. Third, we also had interests in whether respondents used an electronic wearable device to monitor health conditions or activity. All the questions were framed within the time limit of the past 12 months. We coded all the outcomes as binary variables; in order to encompass a large group who fail to use digital health technology due to the lack of internet connection or digital devices, we also coded the respondents who reported no internet access, no digital device, or only basic cell phone as 0 in our outcomes. We believe this full range of digital health technology variables covers the majority of digital health technology that Americans currently rely on to seek health information, access health services, and maintain overall well-being.

#### COVID-19 Pandemic

The pandemic indicator was generated based on when the survey was completed before March 11, 2020. The prepandemic period consisted of the 2017, 2018, 2019, and 2020 prepandemic periods as flagged by HINTS, while we classified 2022 as the postpandemic period.

#### Age Groups

Following the definition of age group in medical subject headings [37], we classified the sample into three age groups based on their self-reported ages: (1) adults (aged 18-44 years), (2) middle-aged adults (aged 45-64 years), and (3) older adults (aged 65 years or older).

#### Control Covariates

Following the existing literature, 3 sets of individual-level control covariates were introduced into our study to alleviate the concerns of confounders in digital health technology uses, which were demographic, socioeconomic, and health [12,22,38,39]. First, the demographic control covariates included race and ethnicity (non-Hispanic White [reference group], Hispanic, non-Hispanic Black, and other non-Hispanic), sex (male and female), marital status, and household size. Second, socioeconomic covariates contained education level (less than high school [reference group], high school, some college, college graduate, or more), household annual income in the past year (less than US $20,000 [reference group]; US $20,000-34,999; US $25,000-49,999; US $50,000-74,999; and US $75,000 or more), residing in a metropolitan area, and census region (Northeast [reference group], Midwest, South, and West). Third, evidence indicates that individuals with different general health status tend to have different degrees of digital health technology use [40], and that adoption of digital health technologies is more prevalent among those with chronic conditions [41], including diabetes [42], hypertension [43], heart conditions [44], chronic lung disease [45], and depression [46] in certain conditions. Therefore, we considered the following variables as health-related control covariates: insured, general health status (poor [reference group], fair, good, very good, and excellent), BMI, diagnosed as diabetes, hypertension, heart conditions, chronic lung disease or depression, and mental health scores derived from a list of mental health issues in the past 2 weeks.

### Analytical Approach

In the descriptive analysis, we computed the descriptive statistics for 6 outcome variables of digital health technology use and the control covariates in the overall sample and 3 subsamples of adults, middle-aged adults, and older adults. Also, we showed the unadjusted trends of digital health technology use across the pandemic by different age groups in the graph.

We used the multivariate LPM, the extension of ordinary least squares regression in the binary outcome, to detect any significant differences in the changes of outcomes due to the pandemic across various age groups. In other words, we compared the pre-post differences in digital health technology use in the target age group, either middle-aged or older adults, with the differences in the reference group (ie, adults). Specifically, we estimated the following equation:




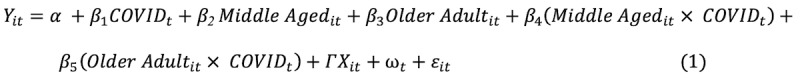




*Y_it_* is the outcome for individual *i* at year *t*. *COVID_t_* is the dummy variable indicating whether the individual was surveyed after the onset of COVID-19 pandemic. *Middle Aged_it_* and *Old Adult_it_* are dummy indicators of age group. *X_it_* captures the individual-level confounders of digital health technology use. *ω_t_* includes year fixed effects to account for common changes in outcomes shared by each survey cohort. *β_1_* reflects the effect of the pandemic on the adults group. *β_2_* is the effect of the middle-aged population before the pandemic, while *β_3_* catches the impact of older adults before the pandemic. Our key parameters of interest are *β_4_* and *β_5_*, which allows us to determine whether the COVID-19 pandemic affected various age groups to different extents. *β_4_* indicates the changes of disparity in digital health technology use between middle-aged adults and adults in the postpandemic period, relative to the prepandemic disparity between middle-aged adults and adults. If the signs of *β_4_* and *β_2_* are the same, the gap between middle-aged adults and adults expands; otherwise, the gap narrows. Similarly, we can interpret *β_5_* as the pre-post changes in the differences between adults and older adults and conclude whether the disparity between adults and older adults is exacerbated by the signs of *β_5_* and *β_3_*.

Because pandemic-related changes of age-based digital disparities may be different across various demographic groups, we conducted several subgroup analyses to differentiate the heterogeneous effects and separately estimated the same regression model shown in equation 1 among multiple groups of race and ethnicity, marital status, sex, and rurality. In addition, the sensitivity analyses included (1) estimating the logit model which is used for the binary outcomes and comparing with the LPM model, (2) various approaches of sample selections for the 2020 sample, and (3) estimating the model in a subset sample with internet connections and digital devices in order to isolate the influence of objective barriers.

All the analyses were conducted on Stata (version 18; StataCorp) [47]. All LPM coefficients were scaled by 100 for readability. To account for the HINTS complex sample design and obtain the nationally representative estimates, we calculated the jackknife replication SE and incorporated sampling weights into descriptive and regression analyses, suggested by HINTS [48] as well as previous studies [12,18,38]. We used the weights provided by HINTS and the Stata survey command “svy” to implement this complex survey weighting process and obtain the weighted statistics. The statistical significance level was set at *P*<.05. 

### Ethical Considerations

Given that HINTS data collection has received approval from the institutional review board, and our secondary data analysis used deidentified, publicly available information, our study is exempt from institutional review board review.

## Results

### Descriptive Analysis

Among the 15,505 survey respondents across 2017-2022, a total of 4361 were adults, 6055 were middle-aged adults, and 5089 were older adults. Table 1 summarizes their digital health technology use patterns and personal characteristics. Overall, US populations tended to use the internet to look for health information (11,529/15,411, 74.81%), followed by using health apps (3073/6059, 50.71%), making appointments (5807/12,771, 45.47%), using the internet to communicate with health providers (6825/15,399, 44.32%), and looking up test results (6649/15,370, 43.26%); the lowest was using electronic wearable devices to track health, with only 32.89% (3394/10,318). Adults were most likely to use digital health technology, while older adults demonstrated the lowest likelihood. Several individual characteristics across age groups are noticeable. Older adults were more likely to report as non-Hispanic White (4011/5089, 78.82%), female (2695/5089, 52.96%), having less than high school or high school education level (1710/5089, 33.6%), and having chronic diseases except for depression (ie, diabetes: 1470/5089, 28.89%; hypertension: 3278/5089, 64.41%; heart conditions: 927/5089, 18.22%; and chronic lung diseases: 795/5089, 15.62%). These differences suggest potential confounders and the necessity of adjusted regression.

**Figure 1:**
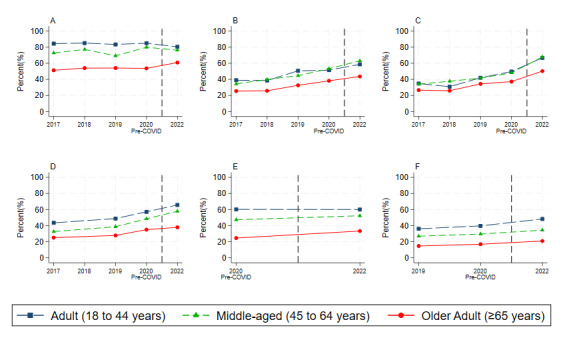
Unadjusted trends of digital health technology use by age groups: (A) look for health information, (B) communicate with health providers, (C) look up test results, (D) make appointments, (E) use health apps, and (F) use wearable devices to track health. All statistics are weighted to reflect the complex sampling strategy of the survey. Source: authors' own analyses of the 2017-2022 Health Information National Trends Survey.

We also visualized the unadjusted trends of digital health technology use in 2017-2022 by various age groups in Figure 1. Compared with adults and middle-aged adults, older adults significantly underutilized digital tools for health-related activities. Entering the COVID-19 pandemic, all 3 groups exhibited increased use of digital health technology to some extent; however, slightly wider gaps between older adults and the other 2 groups were observed regarding communicating with health providers, looking up test results, making appointments, using health apps and using wearable devices to track health. The age-based disparity only became smaller in using the internet to look for health information. Unadjusted regression results in Table S1 in Multimedia Appendix 1 correspond with our observation in Figure 1.

**Table 1:** Characteristics of the sample by age groups. All statistics are weighted to reflect the complex sampling strategy of the survey. Sample size may vary for outcome variables due to missing values. Source: authors’ own analyses of the 2017-2022 Health Information National Trends Survey.

Variables					Full sample (N=15,505)		Adults (18-44 years; n=4361)		Middle-aged (45-64 years; n=6055)		Older adult (≥65 years; n=5089)
**Outcomes, n (%)**											
	Look for health information				11,529 (74.81)		3628 (83.40)		4474 (74.32)		2790 (55.35)
	Communicate with health provider				6825 (44.32)		2058 (47.31)		2763 (45.94)		1680 (33.35)
	Look up test results				6649 (43.26)		1932 (44.52)		2715 (45.20)		1793 (35.68)
	Make appointments				5807 (45.47)		1943 (53.45)		2136 (43.36)		1328 (31.55)
	Use health apps				3073 (50.71)		1064 (60.07)		1115 (50.97)		650 (30.98)
	Use wearable devices to track health				3394 (32.89)		1241 (42.04)		1174 (30.39)		628 (17.91)
**Control covariates**											
	**Demographics**										
		**Race and ethnicity, n (%)**									
			Non-Hispanic White	10,007 (64.54)		2486 (57)		4004 (66.13)		4011 (78.82)	
			Hispanic	2468 (15.92)		884 (20.27)		879 (14.52)		447 (8.79)	
			Non-Hispanic Black	1642 (10.59)		450 (10.31)		736 (12.16)		386 (7.58)	
			Other non-Hispanic	1388 (8.95)		542 (12.42)		435 (7.18)		245 (4.81)	
		**Sex, n (%)**									
			Male	7771 (50.12)		2225 (51.02)		3058 (50.51)		2394 (47.04)	
			Female	7734 (49.88)		2136 (48.98)		2997 (49.49)		2695 (52.96)	
		**Marital status, n (%)**									
			Married	8689 (56.04)		1918 (43.97)		4052 (66.92)		3032 (59.58)	
			Unmarried	6816 (43.96)		2443 (56.03)		2003 (33.08)		2057 (40.42)	
		**Household size, mean (SD)**			3.11 (0.17)		3.77 (0.39)		2.90 (0.03)		2.05 (0.02)
	**Socioeconomic**										
		**Education level, n (%)**									
			Less than high school	1,081 (6.97)		265 (6.07)		440 (7.26)		432 (8.49)	
			High school	3,237 (20.88)		770 (17.66)		1,355 (22.37)		1,278 (25.11)	
			Some college	5,959 (38.43)		1,618 (37.11)		2,387 (39.43)		1,999 (39.28)	
			College graduate or more	5,227 (33.71)		1,708 (39.16)		1,874 (30.95)		1,381 (27.13)	
		**Household annual income, n (%)**									
			Less than US $20,000	2,380 (15.35)		692 (15.86)		850 (14.04)		874 (17.18)	
			US $20,000-US $34,999	1,724 (11.12)		468 (10.73)		550 (9.09)		852 (16.75)	
			US $35,000-US $49,999	2,008 (12.95)		593 (13.59)		651 (10.75)		841 (16.52)	
			US $50,000-US $74,999	2,841 (18.32)		767 (17.58)		1,100 (18.16)		1,043 (20.49)	
			US $75,000 or more	6,552 (42.26)		1,842 (42.24)		2,904 (47.96)		1,479 (29.06)	
		**Rurality, n (%)**									
			Nonmetropolitan	2002 (12.91)		477 (10.94)		812 (13.41)		837 (16.44)	
			Metropolitan area	13,503 (87.09)		3884 (89.06)		5243 (86.59)		4252 (83.56)	
		**Census region, n (%)**									
			Northeast	2741 (17.68)		755 (17.31)		1053 (17.39)		978 (19.21)	
			Midwest	3304 (21.31)		942 (21.61)		1332 (22)		967 (19.01)	
			South	5816 (37.51)		1554 (35.63)		2321 (38.34)		2040 (40.09)	
			West	3644 (23.50)		1109 (25.44)		1348 (22.27)		1103 (21.68)	
	**Health**										
		**Health insurance status, n (%)**									
			Insured	14,105 (90.97)		3765 (86.33)		5594 (92.39)		5024 (98.73)	
			Uninsured	1400 (9.03)		596 (13.67)		461 (7.61)		65 (1.27)	
		**General health status, n (%)**									
			Poor	340 (2.19)		70 (1.61)		150 (2.48)		149 (2.92)	
			Fair	1989 (12.83)		445 (10.21)		831 (13.73)		867 (17.03)	
			Good	5399 (34.82)		1422 (32.61)		2193 (36.22)		1873 (36.80)	
			Very good	5755 (37.12)		1730 (39.68)		2165 (35.75)		1740 (34.19)	
			Excellent	2020 (13.03)		693 (15.89)		715 (11.81)		461 (9.06)	
		**BMI, mean (SD)**			28.62 (0.10)		27.99 (0.20)		29.46 (0.11)		28.17 (0.11)
		**Chronic disease, n (%)**									
			Diabetes	2513 (16.21)		295 (6.76)		1239 (20.47)		1470 (28.89)	
			Hypertension	5462 (35.23)		719 (16.48)		2538 (41.92)		3278 (64.41)	
			Heart conditions	1116 (7.20)		126 (2.88)		418 (6.91)		927 (18.22)	
			Chronic lung diseases	1775 (11.45)		416 (9.54)		703 (11.61)		795 (15.62)	
			Depression	3974 (25.63)		1259 (28.87)		1567 (25.88)		880 (17.29)	
		**Mental health scores, mean (SD)**			13.75 (0.04)		13.33 (0.10)		13.92 (0.06)		14.36 (0.06)
**Observations, n**					15,505		4361		6055		5089

### Regression Results

Table 2 reports coefficients of the COVID-19 indicator, age groups, and their interactive terms in equation 1 accounting for confounders in Table 1. Compared with adults, older adults showed greater reluctance against digital health technology, followed by middle-aged adults. Older adults were 26.28% less likely to use the internet to look for health information (*P*<.001), 13.38% less likely to communicate with health providers through the internet (*P*<.001), 7.63% less likely to use the internet to look up test results (*P*<.001), 14.97% less likely to use internet for making appointments (*P*<.001), 32.63% less likely to use health apps (*P*<.001), and 18.31% less likely to use wearable devices to track health (*P*<.001). Middle-aged adults had a lower probability of using the internet for health information seeking (–10.12%; *P*<.001), health care provider communication (–5.22%; *P*=.008), appointment-making (–9.01%; *P*<.001), and using wearable devices to track health (–9.79%; *P*=.001). The magnitudes are only half of the coefficients of older adults.

When the pandemic started, adults used digital health technology with higher probability: adults’ use of the internet to communicate with health care providers increased by 18.78% (*P*<.001), using internet to look up test results increased by 32.38% (*P*<.001), using internet to make appointments increased by 21.28% (*P*<.001), and using wearable devices to track health increased by 11.4% (*P*<.001). However, there were no significant changes in using health apps (*P*=.64), and there were only marginal increases in using the internet to look for health information (*P*=.07), although not statistically significant.

The gaps between older adults and adults after the onset of the pandemic were significantly wider for using the internet to look up test results (–11.21%; *P*<.001), using internet to make appointments (–10.03%; *P*=.006), and using wearable devices to track health (–8.31%; *P*=.01). Conversely, the gap in using the internet to look for health information narrowed (9.64%; *P*=.002). The gaps between middle-aged adults and adults became narrower in both using the internet to look for health information (6.50%; *P*=.049) and communicate with health providers (7.60%; *P*=.05).

With regard to control covariates in the regression model shown in Table S2 in Multimedia Appendix 1, marital status, education level, household annual income, health insurance status, and depression were positively associated with digital health technology use, while male sex and Hispanic ethnicity were negatively associated.

**Figure 2:**
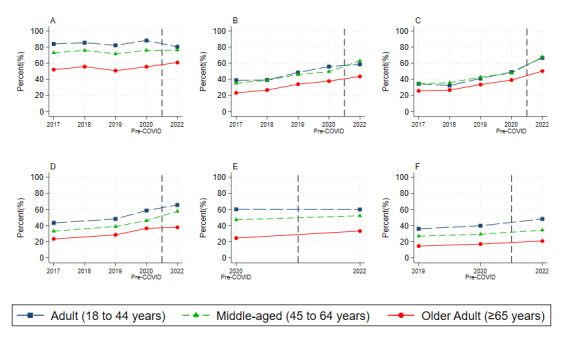
Predicted trends of digital health technology use by age groups: (A) look for health information, (B) communicate with health providers, (C) look up test results, (D) make appointments, (E) use health apps, and (F) use wearable devices to track health. All statistics are weighted to reflect the complex sampling strategy of the survey. Values are predicted by a linear probability model with SE derived from the jackknife replication method. In each regression, we control for the demographic, socioeconomic, and health covariates shown in Table 1, in addition to year fixed effects. Source: authors’ own analyses of the 2017-2022 Health Information National Trends Survey.

The adjusted trends predicted from our regression models were much more interpretable (shown in Figure 2). We made three observations. First, regarding the probability of digital health technology use, the adults ranked highest, followed by middle-aged adults, and older adults ranked the lowest. Second, following the outbreak of the pandemic, we witnessed a prominent growth in digital health technology use among all 3 groups. Third, digital health technology use among adults increased at a more rapid pace than among older adults, so age-based disparities in digital health technology use turned out to widen during the pandemic. However, the disparities were narrowed between middle-aged adults and adults, especially for using the internet to look for health information and communicate with health providers.

**Table 2:** Regressions of COVID-19 pandemic influence on age-based disparities in health technology use. Regression models were estimated by linear probability model regression, and SE derived from the jackknife replication method are shown in parentheses. All linear probability model coefficients were scaled by 100 for readability. In each regression, we controlled for the demographic, socioeconomic, and health covariates shown in in addition to year fixed effects. Source: authors’ own analyses of the 2017-2022 Health Information National Trends Survey.

		Look for health information		Communicate with health providers			Look up test results			Make appointments			Use health apps			Use wearable devices to track health	
		Value	*P* value	Value	*P* value	Value		*P* value	Value		*P* value	Value		*P* value	Value		*P* value
**COVID-19, β (SE)**		–4.46 (2.39)	.07	*18.78*^a^ (3.10)	<.001	*32.38* (2.71)		<.001	*21.28* (3.07)		<.001	2.48 (5.31)		.64	*11.40* (2.96)		<.001
**Age group (reference=adults), β (SE)**																	
	Middle-aged adults	–*10.12* (1.56)	<.001	–*5.22* (1.88)	.008	–*1.55* (1.62)		.34	–*9.01* (2.47)		.001	–12.76 (7.67)		.10	–*9.79* (2.75)		.001
	Older adults	–*26.28* (1.94)	<.001	–*13.38* (2.09)	<.001	–*7.63* (1.89)		<.001	–*14.97* (2.73)		<.001	–*32.63* (7.35)		<.001	–*18.3* (2.48)		<.001
**COVID-19 × age group (reference=adults), β (SE)**																	
	Middle-aged adults	*6.50* (3.23)	.049	*7.60* (3.82)	.05	–0.41 (3.36)		.90	2.39 (3.66)		.52	2.13 (8.07)		.79	–5.48 (3.64)		.14
	Older adults	*9.64* (2.93)	.002	–2.57 (3.62)	.48	–*11.21* (3.32)		<.001	–*10.03* (3.50)		.006	4.58 (7.06)		.52	–*8.31* (3.19)		.01
**Observations, n**		15,411		15,399		15,370			12,771			6,059			10,318		
***R^2^***		.19		.18		.19			.14			.17			.13		

^a^Significant results (*P*<.05) are shown in italics.

### Additional Analysis

We conducted the subgroup analyses based on race and ethnicity, sex, marital status, and rurality. Results shown in Tables S3-S6 in Multimedia Appendix 1 generally support the consistent patterns across various demographic groups except for the rurality, due to the small sample size of nonmetropolitan populations. However, non-White, male, and married older adult groups exhibited greater disparities in using the internet to look up test results and using wearable devices to track health conditions after the pandemic. Meanwhile, among non-White, male, and nonmarried groups, the transitions between the negative main effect (ie, the coefficients of age group) and the positive interactive effect (ie, the coefficients of COVID-19 × age group) were significantly larger than our main estimates, suggesting that the age-based disparities in looking for health information through internet became noticeably smaller in such groups. It indicates the complexity of digital health technology use and the potentially heterogeneous effects of certain demographic factors on digital health technology use among older adults, which should capture the attention of future researchers.

We conducted 3 sensitivity analyses to ensure that our main estimates are robust to model specification and sample selection. First, we tested the robustness of the LPM model relative to the logit model. Although the outcomes were binary, we chose LPM instead of the logit model due to potential concerns of interactive terms estimated by the logit model [49,50]. However, we still estimated the logit model and compared their signs with those of our main estimates, and Table S7 in Multimedia Appendix 1 suggests both estimated effects have the same directions. Second, we were concerned about the issue that the 2020 sample spanned across the outbreak of the pandemic, so we tried 2 approaches, either eliminating the 2020 sample from the regression model (Table S8 in Multimedia Appendix 1) or including the 2020 post-pandemic–onset sample (Table S9 in Multimedia Appendix 1). The results are consistent with our main estimates with slightly different magnitudes. Finally, as the age-based disparity in digital health technology uses results from objective barriers (eg, bad internet connections or no digital devices) [11-13] and subjective barriers such as health literacy [14], we excluded the sample without internet connections or digital devices to isolate the effect of objective barriers. Results in Table S10 in Multimedia Appendix 1 show that the significant effects of the pandemic on reducing age-based disparity among middle-aged adults disappeared but the effects among older adults remained significant and unchanged.

## Discussion

### Principal Findings

Our study explores the pre-post changes of digital health technology use driven by the COVID-19 pandemic among different generations by analyzing the nationally representative cross-sectional sample across 2017-2022. We found that (1) the usage of digital health technology for middle-aged adults and older adults was at disproportionately lower rates than younger cohorts, (2) the usage of digital health technology for all age groups had abruptly increased after the onset of the pandemic, and (3) age-based digital disparities significantly widened between older adults and adults in using the internet to look up test results or make appointments and using wearable devices to track health conditions.

Our study makes significant contributions to the literature on health disparities and the digital divide. There have been debates about whether the perceived health risk associated with the pandemic overpowers the existing objective or psychological disadvantages among vulnerable populations, thereby reducing the age-based disparities in digital health technology use. Using long-period nationwide datasets across the prepandemic era, the onset of the pandemic, and the late pandemic period, we provide the latest empirical evidence showing that age-based disparities expand due to the pandemic, which echoes previous literature that the digital divide is increasing during the pandemic around the globe [17,24,51]. Our study also exhibits the complexities of age-based disparities in digital health technology use, highlighting that the disparities of online health information seeking have narrowed, but disparities of advanced use of digital health technology have widened. Thus, it is needed to delve into the nuanced aspects of digital health technology use in future research.

Compared with the widening age-based disparities in using the internet to look up test results or using wearable devices to track health conditions, the expanding disparities in using the internet to make appointments should raise the attention of policy makers and professionals. Web-based medical appointment system is deemed as an advanced informational technology application with several merits, such as achieving patient-centered services, improving patient satisfaction, curbing no-show rates, and reducing waiting time [52]. In contrast to traditional methods, such as phone calls and in-person visits, web-based appointment systems have higher success rates and reduced waiting times due to after-hour access and enhanced scheduling transparency. However, considering the limited availability of time slots, older adults may be in a disadvantageous position when competing for health care service appointments against younger generations, as they rely more on the traditional appointment approaches as indicated by our findings. Furthermore, the usage of digital health technology is unevenly distributed among older adults [53]: our additional analysis in Table S11 in Multimedia Appendix 1 suggests that education, income, and rurality serve as significant predictors of limited adoption of web-based medical appointment systems in older adult population. Therefore, as the age-based disparity increases over time, older adults, especially those with socioeconomic vulnerability, may encounter restricted access to health care services when needed [54]. As US hospitals enthusiastically adopt digital health care systems [55], it is imperative that health care providers address the needs of older adults who are marginalized and left behind in this digital age.

The only reduced disparity between older adults and younger populations is the disparity in seeking health information through the internet. One factor is the unchanged trend of using the internet for health information among adults. Before the pandemic, we observed the possibility of adults seeking health information online had already reached the maximum level of about 80%, and even the pandemic hardly raised this possibility. Another attribute factor is that the pandemic not only increased the level of perceived risk but also interrupted in-person health care delivery. As older adults had substantial needs for health information, which have been previously addressed through in-person communication with health care providers, they were forced to seek such information online alternatively. Notably, despite the promising trend, only 50% of older adults obtain the necessary health-related information through the internet.

Another important finding of our study is that the often-neglected middle-aged adults, who grow up with digital technology and are supposed to have a higher level of digital literacy than the older adults, are also found to be suffering from lower rates of digital health technology use to some extent. Existing studies point out that not only older adults but also middle-aged adults can hardly use digital tools to access health information [56,57]. Our study shows that middle-aged adults have increasingly used digital health technology for searching for health information and messaging doctors without any significant differences in the adult population, but they still did not substantially use digital health technology to make appointments or track health activities through wearable devices. This means that a large group of middle-aged adults have health care demands and get access to digital health technology, but they still lack instructions and supportive services for using digital health technology to gain effective health care services and achieve better health management. Therefore, digital literacy programs, the design of digital health technology, and digital resources that previously targeted older adults should also take into account middle-aged adults.

In our sensitivity analysis, we tried to eliminate the effect of objective obstacles such as broadband, internet access, or digital devices, but the estimates remained similar, suggesting the change of age-based disparity in digital health technology use results from subjective barriers rather than infrastructure conditions. Scholars have identified several psychological factors of the digital divide, including digital literacy proficiency [58,59], technology anxiety [20,60-62], wariness of cybercrime or privacy violation, or fear of social labels as obsolete [63]. In addition, user-unfriendly interfaces may render low readability of text and usability or apps for older adults [59,64], and these issues might be more serious during the pandemic when digital interventions were intensively introduced without sufficient consultation and evaluation.

### Policy and Practical Implications

During the COVID-19 pandemic, federal and state governments have made substantial efforts to improve internet connectivity [65]. For example, the Affordable Connectivity Program enabled low-income households to afford broadband [66], improved telehealth accessibility, and expanded residential broadband infrastructure. Those endeavors are critical to reach unconnected Americans, especially those in rural, remote, and underserved communities. However, additional approaches and strategies are necessary to address older adults’ psychological concerns related to digital health technology usage as this study indicates.

We propose 3 approaches to increasing digital health technology usage among older adults from the perspectives of local community engagement, technology redesign, and governmental support. First, local community and health care systems could enhance the education programs for digital literacy for older populations. For example, face-to-face training can be delivered at community-based health centers to help older adults install and use digital health technology. Second, more older adult–friendly interfaces or apps should be designed to relieve the operational burdens for such adults to use digital health technology. Researchers start to emphasize the variation of user demands and preferences during the development of digital health tools, and take the needs and preferences of such vulnerable populations into consideration, such as the user-centered design approach [67] or a participatory digital co-design approach [68]. Third, Medicare or state Medicaid programs could consider funding some projects to encourage health care providers to develop and implement interventions aimed at enhancing the use of digital health technology among older adults. For example, projects can invest in robust telehealth infrastructure to assist health care providers in easily reaching out to older adults, launch health care awareness campaigns to educate older adults on the potential benefits of using digital health technologies, and develop health education protocols and offer practical training for health care providers regarding how to communicate with older adults about using digital health tools. These approaches can help ensure these vulnerable populations are not left behind by the digitalized health care system and are adequately prepared for future public health crises.

### Limitations

This study has several limitations. First, this study is a correlation study instead of a causality study. Although we included several control covariates to alleviate the confounder issue, there may be other unobservable confounding factors that bias our estimates, so readers should interpret our findings as associative instead of causal. Second, our analytic sample comes from HINTS collected by the NCI, which inevitably includes the disproportionate older population and oversamples of ethnic minority and rural populations [69]. We have weighted our inference statistics using the sample weights suggested by HINTS, but further research may use other datasets (eg, National Health Interview Survey) to re-examine the changes of age-based disparities and ensure our findings are not distorted by the sampling strategy and selected sample. Third, restricted by the HINTS survey item, we failed to examine the change of frequency of digital health technology use related to the COVID-19 pandemic. Finally, the reasons behind the low use rate for middle-aged adults and older adults are still unclear; thereby, it is difficult to develop detailed approaches for alleviating the age-based disparities. Future studies can undertake comprehensive interviews or detailed surveys to examine the underlying psychological, socioeconomic, and technological factors contributing to the disparities in the use of digital health technology.

### Conclusions

According to the World Health Organization [70], the proportion of global older adults will nearly double from 12% to 22% between 2015 and 2050. Although information technology adoption in the US health care system enhances patient engagement, improves health care outcomes, and increases patient safety, facilitating digital inclusion for the aging baby boomers is still a challenging task. Our study is the first work that systematically examines the changes in age-based disparities in digital health technology use over a long period spanning the pandemic. We find that although the gap in health information seeking through the internet narrowed during the pandemic, being capable of using digital health technology to access health care providers and services remained a challenge. This empirical evidence implies that older adults may be marginalized by the US digitalized health care systems during public health crises such as the COVID-19 pandemic, and therefore health policy makers and health care providers should develop novel approaches to encourage the adoption and use of digital health technology among these vulnerable populations.

## Appendix

Multimedia Appendix 1 Additional information and sensitivity analysis.

## Abbreviations

HINTS: Health Information National Trends Survey
LPM: linear probability model
NCI: National Cancer Institute

## Data Availability

The original datasets can be downloaded from the publicly available Health Information National Trends Survey. The datasets generated during and/or analyzed during this study are available from the corresponding author upon reasonable request.
