# Supplementary material for: The Effects of the COVID-19 Pandemic on Age-Based Disparities in Digital Health Technology Use: Secondary Analysis of the 2017-2022 Health Information National Trends Survey
Source: J Med Internet Res. 2024 Dec 4;26:e65541. doi: 10.2196/65541 (PMC11656112; doi:10.2196/65541)
Supplement: Multimedia Appendix 1 [file jmir_v26i1e65541_app1.docx]

**Table S1. Unadjusted Regressions of COVID-19 Pandemic Influence on Age-based Disparities in Digital Health Technology Use**

|  | **(1)** | | **(2)** | | **(3)** | | **(4)** | | **(5)** | | **(6)** | |
| --- | --- | --- | --- | --- | --- | --- | --- | --- | --- | --- | --- | --- |
|  | **Look for Health Information** | | **Communicate with Health Providers** | | **Look up Test Results** | | **Make Appointments** | | **Use Health Apps** | | **Use Wearable Devices to Track Health** | |
|  | β / SE | *P*-value | β / SE | *P*-value | β / SE | *P*-value | β / SE | *P*-value | β / SE | *P*-value | β / SE | *P*-value |
| COVID | -3.25 | .22 | **20.39** | *<.001* | **33.10** | *<.001* | **22.24** | *<.001* | -0.14 | .98 | **12.06** | *<.001* |
|  | (2.63) |  | (3.39) |  | (2.85) |  | (3.24) |  | (6.22) |  | (3.11) |  |
| Age Group  (Ref.=Adult) |  |  |  |  |  |  |  |  |  |  |  |  |
| *Middle-aged* | **-10.60** | *<.001* | -2.57 | .22 | 1.44 | .44 | **-10.22** | *<.001* | -12.86 | .12 | **-9.35** | .001 |
|  | (1.61) |  | (2.07) |  | (1.84) |  | (2.72) |  | (8.16) |  | (2.76) |  |
| *Older Adult* | **-31.25** | *<.001* | **-14.66** | *<.001* | **-7.60** | *<.001* | **-20.23** | *<.001* | **-35.57** | *<.001* | **-21.78** | *<.001* |
|  | (1.72) |  | (1.99) |  | (1.81) |  | (2.31) |  | (7.49) |  | (2.42) |  |
| COVID × Age Group (Ref.=Adult) |  |  |  |  |  |  |  |  |  |  |  |  |
| *Middle-aged* | 6.41 | .09 | 6.83 | .12 | -0.46 | .90 | 2.39 | .55 | 5.17 | .55 | -4.54 | .26 |
|  | (3.67) |  | (4.33) |  | (3.71) |  | (3.99) |  | (8.61) |  | (3.95) |  |
| *Older Adult* | **11.74** | *<.001* | -0.44 | .91 | **-8.90** | .01 | **-7.58** | .04 | 8.87 | .25 | -5.65 | .09 |
|  | (3.08) |  | (3.99) |  | (3.46) |  | (3.56) |  | (7.60) |  | (3.29) |  |
| **Observations** | 15,411 | | 15,399 | | 15,370 | | 12,771 | | 6,059 | | 10,318 | |
| **R^2^** | .05 | | .04 | | .07 | | .06 | | .05 | | .04 | |

Source: Authors' own analyses of the 2017-2022 Health Information National Trends Survey. Note: Significant results (*P*<.05) are shown in bold. Regression models are estimated by linear probability model (LPM), and standard errors derived from the jackknife replication method are shown in parentheses. All LPM coefficients are scaled by 100 for readability. In each regression, we only control for year fixed effects. Abbreviations: SE=Standard Error; Ref.=Reference.

**Table S2. Full Set of Coefficients in Regressions of COVID-19 Pandemic Influence on Age-based Disparities in Digital Health Technology Use**

|  | **(1)** | | **(2)** | | **(3)** | | **(4)** | | **(5)** | | **(6)** | |  |  |  |  |  |
| --- | --- | --- | --- | --- | --- | --- | --- | --- | --- | --- | --- | --- | --- | --- | --- | --- | --- |
|  | **Look for Health Information** | | **Communicate with Health Providers** | | **Look up Test Results** | | **Make Appointments** | | **Use Health Apps** | | **Use Wearable Devices to Track Health** | |  |  |  |  |  |
|  | β / SE | *P*-value | β / SE | *P*-value | β / SE | *P*-value | β / SE | *P*-value | β / SE | *P*-value | β / SE | *P*-value |  |  |  |  |  |
| COVID | -4.46 | .07 | **18.78** | *<.001* | **32.38** | *<.001* | **21.28** | *<.001* | 2.48 | .64 | **11.40** | *<.001* |  |  |  |  |  |
|  | (2.39) |  | (3.10) |  | (2.71) |  | (3.07) |  | (5.31) |  | (2.96) |  |  |  |  |  |  |
| Age Group (Reference=Adult) |  |  |  |  |  |  |  |  |  |  |  |  |  |  |  |  |  |
| *Middle-aged* | **-10.12** | *<.001* | **-5.22** | .008 | -1.55 | .34 | **-9.01** | .001 | -12.76 | .10 | **-9.79** | .001 |  |  |  |  |  |
|  | (1.56) |  | (1.88) |  | (1.62) |  | (2.47) |  | (7.67) |  | (2.75) |  |  |  |  |  |  |
| *Older Adult* | **-26.28** | *<.001* | **-13.38** | *<.001* | **-7.63** | *<.001* | -**14.97** | *<.001* | **-32.63** | *<.001* | **-18.31** | *<.001* |  |  |  |  |  |
|  | (1.94) |  | (2.09) |  | (1.89) |  | (2.73) |  | (7.35) |  | (2.48) |  |  |  |  |  |  |
| COVID × Age Group (Reference=Adult) |  |  |  |  |  |  |  |  |  |  |  |  |  |  |  |  |  |
| *Middle-aged* | **6.50** | .05 | **7.60** | .05 | -0.41 | .90 | 2.39 | .52 | 2.13 | .79 | -5.48 | .14 |  |  |  |  |  |
|  | (3.23) |  | (3.82) |  | (3.36) |  | (3.66) |  | (8.07) |  | (3.64) |  |  |  |  |  |  |
| *Older Adult* | **9.64** | .002 | -2.57 | .48 | **-11.21** | .001 | **-10.03** | .006 | 4.58 | .52 | **-8.31** | .01 |  |  |  |  |  |
|  | (2.93) |  | (3.62) |  | (3.32) |  | (3.50) |  | (7.06) |  | (3.19) |  |  |  |  |  |  |
| **Control Covariates: Demographic** |  |  |  |  |  |  |  |  |  |  |  |  |  |  |  |  |  |
| Race/Ethnicity (Reference=Non-Hispanic White) |  |  |  |  |  |  |  |  |  |  |  |  |  |  |  |  |  |
| *Hispanic* | **-6.11** | *<.001* | **-7.70** | *<.001* | **-4.74** | .01 | 1.79 | .48 | 1.78 | .63 | 2.06 | .45 |  |  |  |  |  |
|  | (1.63) |  | (1.99) |  | (1.84) |  | (2.53) |  | (3.65) |  | (2.72) |  |  |  |  |  |  |
| *Non-Hispanic Black* | **-4.25** | .02 | -0.14 | .95 | -2.78 | .21 | **7.46** | .009 | -3.21 | .32 | -1.62 | .51 |  |  |  |  |  |
|  | (1.77) |  | (2.19) |  | (2.18) |  | (2.76) |  | (3.18) |  | (2.42) |  |  |  |  |  |  |
| *Other non-Hispanic* | -0.52 | .78 | 1.06 | .73 | 0.71 | .76 | **7.06** | .04 | -0.90 | .83 | 1.19 | .69 |  |  |  |  |  |
|  | (1.85) |  | (3.09) |  | (2.28) |  | (3.38) |  | (4.18) |  | (2.98) |  |  |  |  |  |  |
| Male | **-5.44** | *<.001* | **-6.19** | *<.001* | **-8.47** | *<.001* | **-3.48** | .04 | **-7.44** | .001 | **-8.29** | *<.001* |  |  |  |  |  |
|  | (1.19) |  | (0.94) |  | (1.06) |  | (1.63) |  | (2.13) |  | (1.44) |  |  |  |  |  |  |
| Married | **2.78** | .04 | **3.45** | .009 | **7.43** | *<.001* | 2.15 | .17 | **6.38** | .04 | **4.55** | .009 |  |  |  |  |  |
|  | (1.33) |  | (1.27) |  | (1.58) |  | (1.52) |  | (2.99) |  | (1.67) |  |  |  |  |  |  |
| Household Size | 0.21 | .14 | -**0.42** | .01 | **-0.36** | .001 | -0.29 | .55 | -0.81 | .38 | -0.51 | .29 |  |  |  |  |  |
|  | (0.14) |  | (0.16) |  | (0.10) |  | (0.48) |  | (0.91) |  | (0.48) |  |  |  |  |  |  |
| **Control Covariates: Socioeconomic** |  |  |  |  |  |  |  |  |  |  |  |  |  |  |  |  |  |
| Education Level (Reference=Less than High School) |  |  |  |  |  |  |  |  |  |  |  |  |  |  |  |  |  |
| *High School* | **11.12** | .005 | 1.58 | .60 | 3.86 | .17 | 4.42 | .29 | 2.86 | .57 | -0.02 | .996 |  |  |  |  |  |
|  | (3.83) |  | (2.95) |  | (2.80) |  | (4.16) |  | (4.95) |  | (3.86) |  |  |  |  |  |  |
| *Some College* | **26.21** | *<.001* | **12.18** | .001 | **16.39** | *<.001* | **15.31** | *<.001* | **14.90** | .001 | **8.51** | .04 |  |  |  |  |  |
|  | (3.71) |  | (3.32) |  | (2.74) |  | (3.63) |  | (4.38) |  | (3.95) |  |  |  |  |  |  |
| *College Graduate or More* | **32.98** | *<.001* | **21.80** | *<.001* | **26.13** | *<.001* | **22.93** | *<.001* | **23.91** | *<.001* | **14.03** | .002 |  |  |  |  |  |
|  | (3.65) |  | (3.31) |  | (2.74) |  | (3.92) |  | (5.10) |  | (4.22) |  |  |  |  |  |  |
| Household Annual Income (Reference=Less than $20,000) |  |  |  |  |  |  |  |  |  |  |  |  |  |  |  |  |  |
| *$20,000 to $34,999* | **6.68** | .03 | **5.05** | .02 | 3.19 | .20 | **6.70** | .04 | 5.35 | .18 | 3.16 | .19 |  |  |  |  |  |
|  | (3.04) |  | (2.02) |  | (2.43) |  | (3.14) |  | (3.97) |  | (2.36) |  |  |  |  |  |  |
| *$35,000 to $49,999* | **9.35** | .002 | **9.72** | *<.001* | **7.50** | .003 | **6.63** | .04 | **13.14** | .003 | **9.87** | .003 |  |  |  |  |  |
|  | (2.81) |  | (2.24) |  | (2.37) |  | (3.14) |  | (4.24) |  | (3.11) |  |  |  |  |  |  |
| *$50,000 to $74,999* | **15.77** | *<.001* | **17.57** | *<.001* | **12.48** | *<.001* | **13.20** | *<.001* | **11.79** | .002 | **12.78** | *<.001* |  |  |  |  |  |
|  | (2.72) |  | (2.40) |  | (2.49) |  | (3.32) |  | (3.67) |  | (2.82) |  |  |  |  |  |  |
| *$75,000 or More* | **19.03** | *<.001* | **25.74** | *<.001* | **17.74** | *<.001* | **15.71** | *<.001* | **24.02** | *<.001* | **20.71** | *<.001* |  |  |  |  |  |
|  | (2.56) |  | (2.39) |  | (2.57) |  | (3.21) |  | (4.19) |  | (3.03) |  |  |  |  |  |  |
| Metropolitan Area | **5.10** | .008 | **8.18** | *<.001* | **8.11** | *<.001* | **9.60** | *<.001* | 5.31 | .06 | 3.24 | .17 |  |  |  |  |  |
|  | (1.83) |  | (1.82) |  | (1.54) |  | (2.20) |  | (2.79) |  | (2.31) |  |  |  |  |  |  |
| Census Region (Reference=Northeast) |  |  |  |  |  |  |  |  |  |  |  |  |  |  |  |  |  |
| *Midwest* | 0.41 | .82 | -1.73 | .36 | 3.12 | .09 | 2.73 | .30 | 4.50 | .16 | **5.27** | .02 |  |  |  |  |  |
|  | (1.85) |  | (1.88) |  | (1.78) |  | (2.60) |  | (3.16) |  | (2.24) |  |  |  |  |  |  |
| *South* | **3.67** | .006 | 2.38 | .18 | 1.32 | .47 | **4.78** | .01 | 0.64 | .83 | **4.97** | .04 |  |  |  |  |  |
|  | (1.29) |  | (1.74) |  | (1.80) |  | (1.84) |  | (2.99) |  | (2.35) |  |  |  |  |  |  |
| *West* | 2.93 | .08 | **6.46** | .002 | **7.10** | *<.001* | **8.76** | *<.001* | 1.58 | .67 | 1.32 | .62 |  |  |  |  |  |
|  | (1.66) |  | (1.97) |  | (1.73) |  | (2.02) |  | (3.69) |  | (2.68) |  |  |  |  |  |  |
| **Control Covariates: Health** |  |  |  |  |  |  |  |  |  |  |  |  |  |  |  |  |  |
| Insured | 2.00 | .50 | **12.26** | *<.001* | **11.35** | *<.001* | **9.70** | .01 | **11.21** | .03 | **7.07** | .03 |  |  |  |  |  |
|  | (2.91) |  | (3.18) |  | (2.61) |  | (3.65) |  | (4.90) |  | (3.09) |  |  |  |  |  |  |
| General Health Status (Reference=Poor) |  |  |  |  |  |  |  |  |  |  |  |  |  |  |  |  |  |
| *Fair* | 2.10 | .64 | -7.76 | .07 | -5.39 | .22 | 0.39 | .95 | 3.22 | .58 | -1.22 | .79 |  |  |  |  |  |
|  | (4.45) |  | (4.24) |  | (4.29) |  | (5.87) |  | (5.82) |  | (4.45) |  |  |  |  |  |  |
| *Good* | 3.16 | .44 | -3.72 | .34 | -3.12 | .40 | 3.18 | .55 | 8.34 | .13 | 0.02 | .996 |  |  |  |  |  |
|  | (4.06) |  | (3.89) |  | (3.64) |  | (5.26) |  | (5.48) |  | (4.35) |  |  |  |  |  |  |
| *Very Good* | 4.60 | .33 | -1.06 | .80 | -0.31 | .94 | 10.44 | .08 | 6.47 | .28 | 2.68 | .55 |  |  |  |  |  |
|  | (4.64) |  | (4.16) |  | (3.80) |  | (5.74) |  | (5.87) |  | (4.47) |  |  |  |  |  |  |
| *Excellent* | 1.82 | .70 | -2.51 | .57 | -2.52 | .50 | **11.30** | .03 | 7.85 | .21 | 9.71 | .07 |  |  |  |  |  |
|  | (4.74) |  | (4.44) |  | (3.67) |  | (5.13) |  | (6.24) |  | (5.15) |  |  |  |  |  |  |
| Body Mass Index | 0.13 | .29 | 0.12 | .23 | 0.22 | .06 | 0.17 | .16 | 0.00 | .98 | **0.21** | .04 |  |  |  |  |  |
|  | (0.12) |  | (0.10) |  | (0.11) |  | (0.12) |  | (0.19) |  | (0.10) |  |  |  |  |  |  |
| Chronic Disease |  |  |  |  |  |  |  |  |  |  |  |  |  |  |  |  |  |
| *Diabetes* | -1.12 | .58 | 1.98 | .28 | **4.57** | .01 | 2.17 | .32 | -0.60 | .84 | -3.02 | .18 |  |  |  |  |  |
|  | (1.99) |  | (1.81) |  | (1.72) |  | (2.14) |  | (2.88) |  | (2.23) |  |  |  |  |  |  |
| *Hypertension* | 0.44 | .74 | **3.74** | .01 | 2.12 | .16 | 0.80 | .65 | 4.01 | .08 | 0.56 | .78 |  |  |  |  |  |
|  | (1.32) |  | (1.39) |  | (1.47) |  | (1.75) |  | (2.24) |  | (1.99) |  |  |  |  |  |  |
| *Heart Conditions* | -1.06 | .70 | 2.09 | .49 | 2.43 | .40 | 4.15 | .22 | 0.40 | .91 | 2.93 | .24 |  |  |  |  |  |
|  | (2.74) |  | (3.00) |  | (2.86) |  | (3.35) |  | (3.35) |  | (2.45) |  |  |  |  |  |  |
| *Chronic Lung Disease* | 2.10 | .19 | 1.81 | .40 | 2.96 | .11 | **4.70** | .03 | 5.19 | .08 | 0.18 | .94 |  |  |  |  |  |
|  | (1.57) |  | (2.15) |  | (1.80) |  | (2.04) |  | (2.91) |  | (2.32) |  |  |  |  |  |  |
| *Depression* | **4.49** | .005 | **10.32** | *<.001* | **7.31** | *<.001* | **5.16** | .008 | **7.82** | .005 | 2.03 | .30 |  |  |  |  |  |
|  | (1.52) |  | (1.78) |  | (1.54) |  | (1.86) |  | (2.68) |  | (1.95) |  |  |  |  |  |  |
| Mental Health Scores | -**0.80** | .001 | -0.33 | .22 | 0.07 | .79 | **-1.27** | *<.001* | 0.25 | .57 | -0.19 | .49 |  |  |  |  |  |
|  | (0.22) |  | (0.26) |  | (0.26) |  | (0.30) |  | (0.43) |  | (0.28) |  |  |  |  |  |  |
| Year (Reference=2017) |  |  |  |  |  |  |  |  |  |  |  |  |  |  |  |  |  |
| *2018* | 3.22 | .07 | 3.28 | .09 | 1.08 | .54 | - | - | - | - | - | - |  |  |  |  |  |
|  | (1.75) |  | (1.87) |  | (1.74) |  | - |  | - |  | - |  |  |  |  |  |  |
| *2019* | -1.21 | .40 | **10.89** | *<.001* | **8.27** | *<.001* | **5.14** | .01 | - | - | - | - |  |  |  |  |  |
|  | (1.42) |  | (1.78) |  | (1.70) |  | (1.98) |  | - |  | - |  |  |  |  |  |  |
| *2020* | 1.72 | .46 | **12.91** | *<.001* | **11.84** | *<.001* | **12.16** | *<.001* | - | - | 0.87 | .73 |  |  |  |  |  |
|  | (2.32) |  | (2.57) |  | (2.91) |  | (2.75) |  | - |  | (2.50) |  |  |  |  |  |  |
| Constant | **42.70** | *<.001* | -6.22 | .36 | **-19.16** | .009 | -1.42 | .89 | 0.69 | .96 | -0.06 | .99 |  |  |  |  |  |
|  | (7.83) |  | (6.68) |  | (7.09) |  | (10.35) |  | (13.02) |  | (7.60) |  |  |  |  |  |  |
| **Observations** | 15,411 | | 15,399 | | 15,370 | | 12,771 | | 6,059 | | 10,318 | |  |  |  |  |  |
| **R^2^** | .19 | | .18 | | .19 | | .14 | | .17 | | .13 | |  |  |  |  |  |

Source: Authors' own analyses of the 2017-2022 Health Information National Trends Survey. Note: Significant results (*P*<.05) are shown in bold. Regression models are estimated by linear probability model (LPM), and standard errors derived from the jackknife replication method are shown in parentheses. All LPM coefficients are scaled by 100 for readability. In each regression, we control for demographic, socioeconomic and health covariates shown in Table 1, in addition to year fixed effects. Abbreviations: SE=Standard Error; Ref.=Reference.

**Table S3. Subgroup Analysis of COVID-19 Pandemic Influence on Age-based Disparities in Digital Health Technology Use by Race**

|  | **(1)** | | | | **(2)** | | | | **(3)** | | | | **(4)** | | | | **(5)** | | | | **(6)** | | | |
| --- | --- | --- | --- | --- | --- | --- | --- | --- | --- | --- | --- | --- | --- | --- | --- | --- | --- | --- | --- | --- | --- | --- | --- | --- |
|  | **Look for Health Information** | | | | **Communicate with Health Providers** | | | | **Look up Test Results** | | | | **Make Appointments** | | | | **Use Health Apps** | | | | **Use Wearable Devices to Track Health** | | | |
|  | **Non-White** | | **Non-Hispanic White** | | **Non-White** | | **Non-Hispanic White** | | **Non-White** | | **Non-Hispanic White** | | **Non-White** | | **Non-Hispanic White** | | **Non-White** | | **Non-Hispanic White** | | **Non-White** | | **Non-Hispanic White** | |
|  | **β/SE** | **P-value** | **β/SE** | **P-value** | **β/SE** | **P-value** | **β/SE** | **P-value** | **β/SE** | **P-value** | **β/SE** | **P-value** | **β/SE** | **P-value** | **β/SE** | **P-value** | **β/SE** | **P-value** | **β/SE** | **P-value** | **β/SE** | **P-value** | **β/SE** | **P-value** |
| COVID | **-6.9** | .04 | -2.4 | .47 | **12.7** | .001 | **22.7** | <.001 | **31.0** | <.001 | **33.1** | <.001 | **16.0** | <.001 | **25.6** | <.001 | 2.2 | .81 | 4.3 | .52 | **13.9** | .005 | **9.9** | .01 |
|  | (3.3) |  | (3.3) |  | (3.7) |  | (3.9) |  | (3.5) |  | (4.1) |  | (3.9) |  | (4.4) |  | (8.8) |  | (6.6) |  | (4.7) |  | (3.7) |  |
| Age Group  (Ref.=Adult) |  |  |  |  |  |  |  |  |  |  |  |  |  |  |  |  |  |  |  |  |  |  |  |  |
| *Middle-aged* | **-13.0** | <.001 | **-8.2** | <.001 | **-9.7** | .001 | -2.3 | .34 | -4.8 | .10 | 0.8 | .71 | **-8.9** | .01 | **-8.1** | .009 | -13.3 | .25 | -11.3 | .23 | **-7.8** | .049 | **-1.9** | .004 |
|  | (2.6) |  | (2.0) |  | (2.6) |  | (2.4) |  | (2.9) |  | (2.2) |  | (3.5) |  | (3.0) |  | (11.4) |  | (9.3) |  | (3.9) |  | (3.6) |  |
| *Older Adult* | **-33.2** | <.001 | **-23.1** | <.001 | **-21.4** | <.001 | **-1.1** | <.001 | **-9.0** | .002 | **-6.5** | .01 | **-21.4** | <.001 | **-11.7** | .001 | **-31.2** | .003 | **-32.7** | <.001 | **-15.7** | .001 | **-19.8** | <.001 |
|  | (3.5) |  | (2.1) |  | (2.9) |  | (2.5) |  | (2.8) |  | (2.5) |  | (2.9) |  | (3.3) |  | (1.1) |  | (8.2) |  | (4.5) |  | (3.0) |  |
| COVID × Age Group (Ref.=Adult) | |  |  |  |  |  |  |  |  |  |  |  |  |  |  |  |  |  |  |  |  |  |  |  |
| *Middle-aged* | **11.0** | .03 | 3.5 | .42 | **14.3** | .001 | 2.8 | .55 | 1.3 | .77 | -1.2 | .82 | 4.9 | .30 | -0.7 | .88 | 5.8 | .65 | -1.1 | .91 | -8.1 | .17 | -4.2 | .38 |
|  | (4.8) |  | (4.3) |  | (4.3) |  | (4.6) |  | (4.5) |  | (5.0) |  | (4.7) |  | (4.6) |  | (12.7) |  | (1.0) |  | (5.9) |  | (4.8) |  |
| *Older Adult* | **14.4** | .02 | 6.9 | .07 | 3.5 | .49 | -7.0 | .12 | **-18.1** | <.001 | -9.6 | .05 | -0.2 | .96 | **-16.6** | .002 | 8.3 | .45 | 1.7 | .84 | **-11.7** | .04 | -6.4 | .06 |
|  | (6.0) |  | (3.7) |  | (5.1) |  | (4.5) |  | (4.7) |  | (4.8) |  | (4.8) |  | (5.0) |  | (1.9) |  | (8.4) |  | (5.6) |  | (3.3) |  |
| **Observations** | 5,847 | | 9,564 | | 5,836 | | 9,563 | | 5,824 | | 9,546 | | 4,879 | | 7,892 | | 2,418 | | 3,641 | | 3,992 | | 6,326 | |

Source: Authors' own analyses of the 2017-2022 Health Information National Trends Survey. Note: Significant results (*P*<.05) are shown in bold. Regression models are estimated by linear probability model (LPM), and standard errors derived from the jackknife replication method are shown in parentheses. All LPM coefficients are scaled by 100 for readability. In each regression, we control for demographic, socioeconomic and health covariates shown in Table 1, in addition to year fixed effects. Abbreviations: SE=Standard Error; Ref.=Reference.

**Table S4. Subgroup Analysis of COVID-19 Pandemic Influence on Age-based Disparities in Digital Health Technology Use by Gender**

|  | **(1)** | | | | **(2)** | | | | **(3)** | | | | **(4)** | | | | **(5)** | | | | **(6)** | | | |
| --- | --- | --- | --- | --- | --- | --- | --- | --- | --- | --- | --- | --- | --- | --- | --- | --- | --- | --- | --- | --- | --- | --- | --- | --- |
|  | **Look for Health Information** | | | | **Communicate with Health Providers** | | | | **Look up Test Results** | | | | **Make Appointments** | | | | **Use Health Apps** | | | | **Use Wearable Devices to Track Health** | | | |
|  | **Female** | | **Male** | | **Female** | | **Male** | | **Female** | | **Male** | | **Female** | | **Male** | | **Female** | | **Male** | | **Female** | | **Male** | |
|  | **β/SE** | **P-value** | **β/SE** | **P-value** | **β/SE** | **P-value** | **β/SE** | **P-value** | **β/SE** | **P-value** | **β/SE** | **P-value** | **β/SE** | **P-value** | **β/SE** | **P-value** | **β/SE** | **P-value** | **β/SE** | **P-value** | **β/SE** | **P-value** | **β/SE** | **P-value** |
| COVID | 0.1 | .95 | **-9.4** | .02 | **20.7** | <.001 | **16.6** | <.001 | **32.1** | <.001 | **32.4** | <.001 | **21.9** | <.001 | **20.5** | <.001 | 0.2 | .98 | 5.0 | .60 | **8.7** | .02 | **14.7** | .002 |
|  | (2.2) |  | (3.8) |  | (3.5) |  | (4.1) |  | (3.5) |  | (4.1) |  | (3.9) |  | (5.1) |  | (6.1) |  | (9.6) |  | (3.7) |  | (4.5) |  |
| Age Group  (Ref.=Adult) |  |  |  |  |  |  |  |  |  |  |  |  |  |  |  |  |  |  |  |  |  |  |  |  |
| *Middle-aged* | **-7.0** | <.001 | **-14.0** | <.001 | **-7.5** | <.001 | -3.4 | .27 | -1.5 | .50 | -1.8 | .51 | **-7.6** | .006 | **-10.7** | .006 | **-17.4** | .02 | -8.3 | .54 | **-13.4** | <.001 | -5.9 | .15 |
|  | (1.8) |  | (2.4) |  | (1.9) |  | (3.0) |  | (2.2) |  | (2.8) |  | (2.6) |  | (3.7) |  | (7.3) |  | (13.5) |  | (3.6) |  | (4.0) |  |
| *Older Adult* | **-27.7** | <.001 | **-25.7** | <.001 | **-18.7** | <.001 | **-8.1** | .01 | **-11.0** | <.001 | -4.1 | .16 | **-17.7** | <.001 | **-12.0** | .003 | **-36.2** | <.001 | **-28.5** | .03 | **-19.0** | <.001 | **-17.6** | <.001 |
|  | (2.4) |  | (2.8) |  | (2.4) |  | (3.0) |  | (2.5) |  | (2.8) |  | (3.5) |  | (3.8) |  | (7.8) |  | (12.9) |  | (3.5) |  | (3.7) |  |
| COVID × Age Group (Ref.=Adult) | |  |  |  |  |  |  |  |  |  |  |  |  |  |  |  |  |  |  |  |  |  |  |  |
| *Middle-aged* | 0.8 | .78 | **12.4** | .048 | 1.2 | .76 | **14.4** | .01 | -2.9 | .45 | 2.6 | .64 | -1.3 | .72 | 6.6 | .29 | 4.6 | .59 | -0.2 | .99 | 2.0 | .70 | **-14.0** | .02 |
|  | (2.8) |  | (6.1) |  | (4.1) |  | (5.5) |  | (3.9) |  | (5.5) |  | (3.7) |  | (6.1) |  | (8.5) |  | (13.0) |  | (5.2) |  | (5.6) |  |
| *Older Adult* | **9.9** | .003 | **8.8** | .04 | -2.6 | .53 | -2.9 | .58 | -7.9 | .06 | **-14.6** | .002 | **-9.9** | .04 | **-10.1** | .04 | 6.3 | .40 | 2.4 | .84 | -4.2 | .37 | **-14.2** | .003 |
|  | (3.2) |  | (4.2) |  | (4.1) |  | (5.2) |  | (4.1) |  | (4.6) |  | (4.6) |  | (4.8) |  | (7.5) |  | (11.9) |  | (4.6) |  | (4.5) |  |
| **Observations** | 8,952 | | 6,459 | | 8,944 | | 6,455 | | 8,929 | | 6,441 | | 7,414 | | 5,357 | | 3,580 | | 2,479 | | 5,990 | | 4,328 | |

Source: Authors' own analyses of the 2017-2022 Health Information National Trends Survey. Note: Significant results (*P*<.05) are shown in bold. Regression models are estimated by linear probability model (LPM), and standard errors derived from the jackknife replication method are shown in parentheses. All LPM coefficients are scaled by 100 for readability. In each regression, we control for demographic, socioeconomic and health covariates shown in Table 1, in addition to year fixed effects. Abbreviations: SE=Standard Error; Ref.=Reference.

**Table S5. Subgroup Analysis of COVID-19 Pandemic Influence on Age-based Disparities in Digital Health Technology Use by Marital Status**

|  | **(1)** | | | | **(2)** | | | | **(3)** | | | | **(4)** | | | | **(5)** | | | | **(6)** | | | |
| --- | --- | --- | --- | --- | --- | --- | --- | --- | --- | --- | --- | --- | --- | --- | --- | --- | --- | --- | --- | --- | --- | --- | --- | --- |
|  | **Look for Health Information** | | | | **Communicate with Health Providers** | | | | **Look up Test Results** | | | | **Make Appointments** | | | | **Use Health Apps** | | | | **Use Wearable Devices to Track Health** | | | |
|  | **Non-married** | | **Married** | | **Non-married** | | **Married** | | **Non-married** | | **Married** | | **Non-married** | | **Married** | | **Non-married** | | **Married** | | **Non-married** | | **Married** | |
|  | **β/SE** | **P-value** | **β/SE** | **P-value** | **β/SE** | **P-value** | **β/SE** | **P-value** | **β/SE** | **P-value** | **β/SE** | **P-value** | **β/SE** | **P-value** | **β/SE** | **P-value** | **β/SE** | **P-value** | **β/SE** | **P-value** | **β/SE** | **P-value** | **β/SE** | **P-value** |
| COVID | -3.8 | .31 | -3.9 | .11 | **18.6** | <.001 | **19.7** | <.001 | **34.6** | <.001 | **31.2** | <.001 | **23.3** | <.001 | **20.0** | <.001 | -3.5 | .65 | 8.5 | .17 | 7.0 | .17 | **16.9** | <.001 |
|  | (3.7) |  | (2.4) |  | (4.2) |  | (3.7) |  | (4.2) |  | (3.1) |  | (5.2) |  | (3.5) |  | (7.8) |  | (6.1) |  | (5.0) |  | (3.2) |  |
| Age Group  (Ref.=Adult) |  |  |  |  |  |  |  |  |  |  |  |  |  |  |  |  |  |  |  |  |  |  |  |  |
| *Middle-aged* | **-9.3** | .003 | **-9.8** | <.001 | -5.5 | .06 | -4.4 | .10 | -1.5 | .59 | -1.4 | .48 | -8.4 | .06 | **-7.7** | .005 | -20.4 | .09 | -7.6 | .35 | -9.6 | .06 | **-9.0** | .001 |
|  | (2.9) |  | (1.8) |  | (2.9) |  | (2.6) |  | (2.8) |  | (2.0) |  | (4.3) |  | (2.6) |  | (11.7) |  | (8.1) |  | (5.1) |  | (2.6) |  |
| *Older Adult* | **-33.2** | <.001 | **-21.6** | <.001 | **-17.2** | <.001 | **-9.8** | .001 | **-11.4** | <.001 | **-5.6** | .04 | **-19.6** | <.001 | **-10.1** | <.001 | **-41.8** | <.001 | **-26.5** | .003 | **-16.4** | <.001 | **-18.2** | <.001 |
|  | (3.2) |  | (2.4) |  | (3.4) |  | (2.7) |  | (2.9) |  | (2.6) |  | (4.5) |  | (2.7) |  | (11.0) |  | (8.5) |  | (4.3) |  | (2.6) |  |
| COVID × Age Group (Ref.=Adult) | |  |  |  |  |  |  |  |  |  |  |  |  |  |  |  |  |  |  |  |  |  |  |  |
| *Middle-aged* | 6.3 | .16 | 4.7 | .21 | **11.8** | .02 | 3.8 | .42 | -1.2 | .82 | -0.5 | .88 | -3.3 | .58 | 4.3 | .31 | 8.7 | .49 | -3.8 | .66 | -3.8 | .54 | **-10.1** | .02 |
|  | (4.4) |  | (3.7) |  | (4.9) |  | (4.7) |  | (5.3) |  | (3.6) |  | (5.8) |  | (4.2) |  | (12.3) |  | (8.5) |  | (6.1) |  | (4.1) |  |
| *Older Adult* | **14.3** | .001 | 5.0 | .15 | 1.5 | .74 | -6.5 | .16 | **-10.9** | .02 | **-11.9** | .004 | -8.5 | .06 | **-11.6** | .01 | 12.0 | .23 | -1.4 | .87 | -8.0 | .11 | **-10.8** | .003 |
|  | (4.0) |  | (3.4) |  | (4.3) |  | (4.5) |  | (4.6) |  | (3.9) |  | (4.3) |  | (4.4) |  | (9.9) |  | (8.4) |  | (4.9) |  | (3.5) |  |
| **Observations** | 6,993 | | 8,418 | | 6,989 | | 8,410 | | 6,973 | | 8,397 | | 5,749 | | 7,022 | | 2,802 | | 3,257 | | 4,707 | | 5,611 | |

Source: Authors' own analyses of the 2017-2022 Health Information National Trends Survey. Note: Significant results (*P*<.05) are shown in bold. Regression models are estimated by linear probability model (LPM), and standard errors derived from the jackknife replication method are shown in parentheses. All LPM coefficients are scaled by 100 for readability. In each regression, we control for demographic, socioeconomic and health covariates shown in Table 1, in addition to year fixed effects. Abbreviations: SE=Standard Error; Ref.=Reference.

**Table S6. Subgroup Analysis of COVID-19 Pandemic Influence on Age-based Disparities in Digital Health Technology Use by Rurality**

|  | **(1)** | | | | **(2)** | | | | **(3)** | | | | **(4)** | | | | **(5)** | | | | **(6)** | | | |
| --- | --- | --- | --- | --- | --- | --- | --- | --- | --- | --- | --- | --- | --- | --- | --- | --- | --- | --- | --- | --- | --- | --- | --- | --- |
|  | **Look for Health Information** | | | | **Communicate with Health Providers** | | | | **Look up Test Results** | | | | **Make Appointments** | | | | **Use Health Apps** | | | | **Use Wearable Devices to Track Health** | | | |
|  | **Non-metropolitan** | | **Metropolitan** | | **Non-metropolitan** | | **Metropolitan** | | **Non-metropolitan** | | **Metropolitan** | | **Non-metropolitan** | | **Metropolitan** | | **Non-metropolitan** | | **Metropolitan** | | **Non-metropolitan** | | **Metropolitan** | |
|  | **β/SE** | **P-value** | **β/SE** | **P-value** | **β/SE** | **P-value** | **β/SE** | **P-value** | **β/SE** | **P-value** | **β/SE** | **P-value** | **β/SE** | **P-value** | **β/SE** | **P-value** | **β/SE** | **P-value** | **β/SE** | **P-value** | **β/SE** | **P-value** | **β/SE** | **P-value** |
| COVID | -0.7 | .93 | **-4.9** | .046 | 11.1 | .20 | **19.6** | <.001 | **29.7** | .001 | **32.7** | <.001 | -3.4 | .75 | **24.4** | <.001 | 14.3 | .38 | 0.8 | .89 | 10.5 | .31 | **11.8** | <.001 |
|  | (7.8) |  | (2.4) |  | (8.5) |  | (3.2) |  | (8.3) |  | (2.9) |  | (10.3) |  | (3.1) |  | (16.0) |  | (5.6) |  | (10.2) |  | (3.0) |  |
| Age Group  (Ref.=Adult) |  |  |  |  |  |  |  |  |  |  |  |  |  |  |  |  |  |  |  |  |  |  |  |  |
| *Middle-aged* | **-14.4** | .005 | **-9.2** | <.001 | **-10.9** | .02 | **-4.6** | .03 | -3.2 | .49 | -1.2 | .46 | **-21.9** | .001 | **-7.3** | .005 | 1.3 | .94 | **-14.8** | .07 | -13.6 | .06 | **-9.4** | .001 |
|  | (4.9) |  | (1.5) |  | (4.4) |  | (2.0) |  | (4.6) |  | (1.6) |  | (6.5) |  | (2.5) |  | (18.1) |  | (8.1) |  | (7.2) |  | (2.7) |  |
| *Older Adult* | **-23.9** | .001 | **-26.3** | <.001 | **-12.7** | .02 | **-13.6** | <.001 | -4.6 | .35 | **-8.0** | <.001 | **-21.7** | .004 | **-14.3** | <.001 | -21.4 | .18 | **-34.0** | <.001 | **-21.0** | .005 | **-18.0** | <.001 |
|  | (6.5) |  | (2.0) |  | (5.1) |  | (2.2) |  | (4.9) |  | (2.0) |  | (7.2) |  | (2.8) |  | (15.6) |  | (8.5) |  | (7.2) |  | (2.6) |  |
| COVID × Age Group (Ref.=Adult) | |  |  |  |  |  |  |  |  |  |  |  |  |  |  |  |  |  |  |  |  |  |  |  |
| *Middle-aged* | 13.6 | .12 | 5.4 | .13 | **21.1** | .050 | 5.9 | .14 | 1.0 | .91 | -0.6 | .86 | **24.1** | .04 | -0.1 | .98 | -13.0 | .53 | 3.9 | .65 | -3.0 | .81 | -6.1 | .11 |
|  | (8.6) |  | (3.4) |  | (10.5) |  | (3.9) |  | (9.2) |  | (3.6) |  | (11.5) |  | (3.7) |  | (20.5) |  | (8.5) |  | (12.1) |  | (3.7) |  |
| *Older Adult* | 6.0 | .43 | **10.2** | .001 | -1.5 | .87 | -2.1 | .57 | -15.1 | .12 | **-10.2** | .006 | 3.9 | .71 | **-11.1** | .003 | -11.5 | .53 | 6.5 | .42 | -11.4 | .30 | **-8.2** | .02 |
|  | (7.6) |  | (3.0) |  | (9.0) |  | (3.7) |  | (9.6) |  | (3.6) |  | (10.5) |  | (3.6) |  | (18.2) |  | (8.0) |  | (10.8) |  | (3.4) |  |
| **Observations** | 1,899 | | 13,512 | | 1,896 | | 13,503 | | 1,893 | | 13,477 | | 1,547 | | 11,224 | | 771 | | 5,288 | | 1,231 | | 9,087 | |

Source: Authors' own analyses of the 2017-2022 Health Information National Trends Survey. Note: Significant results (*P*<.05) are shown in bold. Regression models are estimated by linear probability model (LPM), and standard errors derived from the jackknife replication method are shown in parentheses. All LPM coefficients are scaled by 100 for readability. In each regression, we control for demographic, socioeconomic and health covariates shown in Table 1, in addition to year fixed effects. Abbreviations: SE=Standard Error; Ref.=Reference.

**Table S7. Logit Regressions of COVID-19 Pandemic Influence on Age-based Disparities in Digital Health Technology Use**

|  | **(1)** | | **(2)** | | **(3)** | | **(4)** | | **(5)** | | **(6)** | |
| --- | --- | --- | --- | --- | --- | --- | --- | --- | --- | --- | --- | --- |
|  | **Look for Health Information** | | **Communicate with Health Providers** | | **Look up Test Results** | | **Make Appointments** | | **Use Health Apps** | | **Use Wearable Devices to Track Health** | |
|  | β / SE | *P*-value | β / SE | *P*-value | β / SE | *P*-value | β / SE | *P*-value | β / SE | *P*-value | β / SE | *P*-value |
| COVID | -0.32 | .08 | **0.91** | *<.001* | **1.59** | *<.001* | **0.98** | *<.001* | 0.13 | .61 | **0.53** | *<.001* |
|  | (0.18) |  | (0.15) |  | (0.15) |  | (0.15) |  | (0.25) |  | (0.14) |  |
| Age Group  (Ref.=Adult) |  |  |  |  |  |  |  |  |  |  |  |  |
| *Middle-aged* | **-0.73** | *<.001* | **-0.26** | .006 | -0.09 | .25 | **-0.41** | .001 | -0.59 | .11 | **-0.49** | .001 |
|  | (0.12) |  | (0.09) |  | (0.08) |  | (0.11) |  | (0.36) |  | (0.14) |  |
| *Older Adult* | **-1.55** | *<.001* | **-0.69** | *<.001* | **-0.41** | *<.001* | **-0.72** | *<.001* | **-1.57** | *<.001* | **-1.07** | *<.001* |
|  | (0.14) |  | (0.10) |  | (0.10) |  | (0.13) |  | (0.37) |  | (0.13) |  |
| COVID × Age Group (Ref.=Adult) |  |  |  |  |  |  |  |  |  |  |  |  |
| *Middle-aged* | **0.46** | .047 | 0.36 | .07 | -0.04 | .82 | 0.09 | .59 | 0.08 | .84 | -0.22 | .22 |
|  | (0.23) |  | (0.19) |  | (0.18) |  | (0.17) |  | (0.38) |  | (0.18) |  |
| *Older Adult* | **0.57** | .005 | -0.09 | .60 | **-0.56** | .002 | **-0.41** | .02 | 0.25 | .49 | -0.26 | .12 |
|  | (0.19) |  | (0.18) |  | (0.17) |  | (0.17) |  | (0.36) |  | (0.17) |  |
| **Observations** | 15,411 | | 15,399 | | 15,370 | | 12,771 | | 6,059 | | 10,318 | |

Source: Authors' own analyses of the 2017-2022 Health Information National Trends Survey. Note: Significant results (*P*<.05) are shown in bold. Regression models are estimated by logistic regression, and standard errors derived from the jackknife replication method are shown in parentheses. In each regression, we control for demographic, socioeconomic and health covariates shown in Table 1, in addition to year fixed effects. Abbreviations: SE=Standard Error; Ref.=Reference.

**Table S8. Regressions of COVID-19 Pandemic Influence on Age-based Disparities in Digital Health Technology Use Without 2020 Sample**

|  | **(1)** | | **(2)** | | **(3)** | | **(4)** | | **(5)** | | **(6)** | |
| --- | --- | --- | --- | --- | --- | --- | --- | --- | --- | --- | --- | --- |
|  | **Look for Health Information** | | **Communicate with Health Providers** | | **Look up Test Results** | | **Make Appointments** | | **Use Health Apps** | | **Use Wearable Devices to Track Health** | |
|  | β / SE | *P*-value | β / SE | *P*-value | β / SE | *P*-value | β / SE | *P*-value | β / SE | *P*-value | β / SE | *P*-value |
| COVID | -4.73 | .06 | **18.23** | *<.001* | **32.51** | *<.001* | **20.94** | *<.001* | - | - | **11.29** | 0.001 |
|  | (2.44) |  | (3.19) |  | (2.77) |  | (3.18) |  | - |  | (3.17) |  |
| Age Group (Ref.=Adult) |  |  |  |  |  |  |  |  |  |  |  |  |
| *Middle-aged* | **-11.27** | *<.001* | **-6.47** | .003 | -1.31 | .46 | **-9.97** | *<.001* | - | - | **-9.90** | 0.001 |
|  | (1.66) |  | (2.09) |  | (1.74) |  | (2.65) |  | - |  | (2.87) |  |
| *Older Adult* | **-26.90** | *<.001* | **-14.28** | *<.001* | **-6.91** | *<.001* | **-15.18** | *<.001* | - | - | **-17.64** | 0.000 |
|  | (2.12) |  | (2.23) |  | (1.77) |  | (2.77) |  | - |  | (2.44) |  |
| COVID × Age Group (Ref.=Adult) |  |  |  |  |  |  |  |  |  |  |  |  |
| *Middle-aged* | **7.30** | .03 | **8.63** | .04 | -0.46 | .89 | 3.19 | .42 | - | - | -5.26 | 0.176 |
|  | (3.28) |  | (3.99) |  | (3.41) |  | (3.92) |  | - |  | (3.84) |  |
| *Older Adult* | **9.58** | .004 | -2.04 | .57 | **-11.66** | .001 | **-10.08** | .006 | - | - | **-8.43** | 0.020 |
|  | (3.14) |  | (3.59) |  | (3.17) |  | (3.53) |  | - |  | (3.50) |  |
| **Observations** | 14,262 | | 14,253 | | 14,225 | | 11,626 | | - | | 9,174 | |
| **R^2^** | .19 | | .19 | | .20 | | .14 | | - | | .14 | |

Source: Authors' own analyses of the 2017-2022 Health Information National Trends Survey. Note: Significant results (*P*<.05) are shown in bold. Regression models are estimated by linear probability model (LPM), and standard errors derived from the jackknife replication method are shown in parentheses. All LPM coefficients are scaled by 100 for readability. In each regression, we control for demographic, socioeconomic and health covariates shown in Table 1, in addition to year fixed effects. The coefficients of Model 5 in Panel A are void: Because the outcome of using health Apps only appears in the 2020 & 2022 survey, there are no pre period in such model and the focus coefficients become unable to estimate if we exclude the 2020 sample. Abbreviations: SE=Standard Error; Ref.=Reference.

**Table S9. Regressions of COVID-19 Pandemic Influence on Age-based Disparities in Digital Health Technology Use With 2020 Post-pandemic-onset Sample**

|  | **(1)** | | **(2)** | | **(3)** | | **(4)** | | **(5)** | | **(6)** | |
| --- | --- | --- | --- | --- | --- | --- | --- | --- | --- | --- | --- | --- |
|  | **Look for Health Information** | | **Communicate with Health Providers** | | **Look up Test Results** | | **Make Appointments** | | **Use Health Apps** | | **Use Wearable Devices to Track Health** | |
|  | β / SE | *P*-value | β / SE | *P*-value | β / SE | *P*-value | β / SE | *P*-value | β / SE | *P*-value | β / SE | *P*-value |
| COVID | **-8.98** | .001 | -3.10 | .39 | -4.02 | .199 | -2.19 | .56 | -2.45 | .66 | 3.18 | .38 |
|  | (2.54) |  | (3.55) |  | (3.09) |  | (3.71) |  | (5.61) |  | (3.55) |  |
| Age Group (Ref.=Adult) |  |  |  |  |  |  |  |  |  |  |  |  |
| *Middle-aged* | **-10.48** | *<.001* | **-5.31** | .008 | -1.89 | .26 | **-9.54** | *<.001* | -12.36 | .12 | **-10.00** | .001 |
|  | (1.52) |  | (1.91) |  | (1.64) |  | (2.43) |  | (7.76) |  | (2.69) |  |
| *Older Adult* | **-26.83** | *<.001* | **-13.87** | *<.001* | **-8.03** | *<.001* | **-15.62** | *<.001* | **-32.62** | *<.001* | **-18.85** | *<.001* |
|  | (1.94) |  | (2.02) |  | (1.97) |  | (2.66) |  | (7.11) |  | (2.40) |  |
| COVID × Age Group (Ref.=Adult) |  |  |  |  |  |  |  |  |  |  |  |  |
| *Middle-aged* | **5.41** | .04 | 5.92 | .08 | 0.65 | .81 | 3.07 | .35 | -0.36 | .97 | -2.78 | .44 |
|  | (2.57) |  | (3.34) |  | (2.75) |  | (3.25) |  | (8.08) |  | (3.58) |  |
| *Older Adult* | **9.09** | .001 | -0.02 | .99 | **-5.32** | .04 | -6.08 | .07 | 2.92 | .67 | **-5.76** | .05 |
|  | (2.51) |  | (3.03) |  | (2.45) |  | (3.27) |  | (6.87) |  | (2.87) |  |
| **Observations** | 17,295 | | 17,283 | | 17,251 | | 14,654 | | 7,834 | | 12,192 | |
| **R^2^** | .19 | | .17 | | .19 | | .14 | | .17 | | .12 | |

Source: Authors' own analyses of the 2017-2022 Health Information National Trends Survey. Note: Significant results (*P*<.05) are shown in bold. Regression models are estimated by linear probability model (LPM), and standard errors derived from the jackknife replication method are shown in parentheses. All LPM coefficients are scaled by 100 for readability. In each regression, we control for demographic, socioeconomic and health covariates shown in Table 1, in addition to year fixed effects. Abbreviations: SE=Standard Error; Ref.=Reference.

**Table S10. Regressions of COVID-19 Pandemic Influence on Age-based Disparities in Digital Health Technology Use in Sub-Sample with Internet Access and Electronic Devices**

|  | **(1)** | | **(2)** | | **(3)** | | **(4)** | | **(5)** | | **(6)** | |
| --- | --- | --- | --- | --- | --- | --- | --- | --- | --- | --- | --- | --- |
|  | **Look for Health Information** | | **Communicate with Health Providers** | | **Look up Test Results** | | **Make Appointments** | | **Use Health Apps** | | **Use Wearable Devices to Track Health** | |
|  | β / SE | *P*-value | β / SE | *P*-value | β / SE | *P*-value | β / SE | *P*-value | β / SE | *P*-value | β / SE | *P*-value |
| COVID | -4.11 | .10 | **21.2** | *<.001* | **36.4** | *<.001* | **24.1** | *<.001* | 4.93 | .37 | **12.1** | *<.001* |
|  | (2.44) |  | (3.38) |  | (2.95) |  | (3.31) |  | (5.41) |  | (3.16) |  |
| Age Group (Ref.=Adult) |  |  |  |  |  |  |  |  |  |  |  |  |
| *Middle-aged* | **-4.72** | *<.001* | -2.40 | .25 | 1.43 | .41 | **-6.45** | .01 | -10.8 | .18 | **-9.19** | .003 |
|  | (1.09) |  | (2.05) |  | (1.71) |  | (2.48) |  | (7.94) |  | (2.90) |  |
| *Older Adult* | **-10.3** | *<.001* | -4.92 | .07 | -0.22 | .93 | -6.16 | .06 | **-26.1** | .005 | **-17.6** | *<.001* |
|  | (1.60) |  | (2.65) |  | (2.36) |  | (3.25) |  | (8.92) |  | (2.62) |  |
| COVID × Age Group (Ref.=Adult) |  |  |  |  |  |  |  |  |  |  |  |  |
| *Middle-aged* | 2.02 | .53 | 6.26 | .15 | -2.14 | .53 | 0.78 | .84 | 0.35 | .97 | -6.48 | .11 |
|  | (3.17) |  | (4.31) |  | (3.41) |  | (3.90) |  | (8.34) |  | (3.96) |  |
| *Older Adult* | 4.40 | .08 | -3.50 | .37 | **-9.90** | .007 | **-12.7** | .004 | 2.97 | .73 | **-8.43** | .01 |
|  | (2.50) |  | (3.86) |  | (3.53) |  | (4.27) |  | (8.59) |  | (3.29) |  |
| **Observations** | 12,344 | | 12,332 | | 12,307 | | 10,301 | | 5,005 | | 8,425 | |
| **R^2^** | .07 | | .14 | | .17 | | .10 | | .11 | | .10 | |

Source: Authors' own analyses of the 2017-2022 Health Information National Trends Survey. Note: Significant results (*P*<.05) are shown in bold. Regression models are estimated by linear probability model (LPM), and standard errors derived from the jackknife replication method are shown in parentheses. All LPM coefficients are scaled by 100 for readability. In each regression, we control for demographic, socioeconomic and health covariates shown in Table 1, in addition to year fixed effects. Abbreviations: SE=Standard Error; Ref.=Reference.

**Table S11. Regressions of Whether not to Make Appointments via the Internet on Control Covariates in the Older Adult Population**

|  | **Not Make Appointments** | |
| --- | --- | --- |
|  | β / SE | *P*-value |
| **Control Covariates: Demographic** |  |  |
| Race/Ethnicity (Reference=Non-Hispanic White) |  |  |
| *Hispanic* | 1.75 | .51 |
|  | (2.64) |  |
| *Non-Hispanic Black* | -2.48 | .46 |
|  | (3.32) |  |
| *Other* | **-8.35** | .02 |
|  | (3.54) |  |
| Male | -0.26 | .89 |
|  | (1.85) |  |
| Married | -3.18 | .15 |
|  | (2.19) |  |
| Household Size | 1.27 | .29 |
|  | (1.18) |  |
| **Control Covariates: Socioeconomic** |  |  |
| Education Level (Reference=Less than High School) |  |  |
| *High School* | **-11.49** | *<.001* |
|  | (3.00) |  |
| *Some College* | **-19.39** | *<.001* |
|  | (3.12) |  |
| *College Graduate or More* | **-27.25** | *<.001* |
|  | (2.79) |  |
| Household Annual Income (Reference=Less than $20,000) |  |  |
| *$20,000 to $34,999* | -3.33 | .24 |
|  | (2.77) |  |
| *$35,000 to $49,999* | -5.29 | .10 |
|  | (3.15) |  |
| *$50,000 to $74,999* | **-15.81** | *<.001* |
|  | (3.37) |  |
| *$75,000 or More* | **-22.33** | *<.001* |
|  | (3.47) |  |
| Metropolitan Area | **-9.00** | *<.001* |
|  | (2.30) |  |
| Census Region (Reference=Northeast) |  |  |
| *Midwest* | -2.60 | .27 |
|  | (2.35) |  |
| *South* | -5.04 | .07 |
|  | (2.66) |  |
| *West* | **-11.26** | *<.001* |
|  | (2.77) |  |
| **Control Covariates: Health** |  |  |
| Insured | 0.45 | .97 |
|  | (10.40) |  |
| General Health Status (Reference=Poor) |  |  |
| *Fair* | -2.53 | .65 |
|  | (5.50) |  |
| *Good* | -6.31 | .24 |
|  | (5.28) |  |
| *Very Good* | -9.92 | .06 |
|  | (5.18) |  |
| *Excellent* | -10.12 | .12 |
|  | (6.72) |  |
| Body Mass Index | -0.12 | .53 |
|  | (0.19) |  |
| Chronic Disease |  |  |
| *Diabetes* | 1.29 | .59 |
|  | (2.40) |  |
| *Hypertension* | -1.93 | .35 |
|  | (2.06) |  |
| *Heart Conditions* | -1.32 | .59 |
|  | (2.44) |  |
| *Chronic Lung Disease* | -3.24 | .25 |
|  | (2.81) |  |
| *Depression* | **-6.56** | .02 |
|  | (2.70) |  |
| Mental Health Scores | 0.29 | .48 |
|  | (0.40) |  |
| Year (Reference=2017) |  |  |
| *2019* | -2.86 | .20 |
|  | (2.19) |  |
| *2020* | **-7.91** | .02 |
|  | (3.17) |  |
| *2022* | **-9.10** | .001 |
|  | (2.54) |  |
| Constant | **123.71** | *<.001* |
|  | (14.87) |  |
| **Observations** | 4,209 | |
| **R^2^** | .14 | |

Source: Authors' own analyses of the 2017-2022 Health Information National Trends Survey. Note: Significant results (P<.05) are shown in bold. Regression models are estimated by linear probability model (LPM), and standard errors derived from the jackknife replication method are shown in parentheses. All LPM coefficients are scaled by 100 for readability. Abbreviations: SE=Standard Error; Ref.=Reference.
